# Risk factors of hepatitis B virus surface antigen carriage and serological profile of HBsAg carriers in Lomé Togo, 2016

**DOI:** 10.1186/s12889-018-6320-x

**Published:** 2019-01-08

**Authors:** Wemboo Afiwa Halatoko, Akouda Patassi, Pauline Yanogo, Leou Ismael Banla, Adjaho Koba, Zoulkaneiri Issa, Koffi Akolly, Agballa Mébiny-Esso Tchalla, Hamadi Assane, Aboukerim Naba-Mouchedou, Bernard Sawadogo, Simon Nouvura Antara, Kossi Badziklou, Abiba Kere Banla, Didier Koumavi Ekouevi, Idrissa Sanou

**Affiliations:** 1 Institut National d’Hygiène de Lomé, Lome, Togo; 2 Centre Hospitalo-Universitaire Sylvanus Olympio de Lomé, Lome, Togo; 3 Faculté des Sciences de la Santé, Université de Lomé, Lome, Togo; 4 West Africa Field Epidemiology Training Program (WAFETP), Lome, Togo; 5 Department of Microbiology and Immunology, Medical College of Wisconsin, Milwaukee, WI USA; 6 Direction Préfectorale de la Santé de Haho, Notse, Togo; 7 Division de la Surveillance Intégrée et de Urgences Sanitaires, Lome, Togo; 8 Division de la Santé Communautaire et des Personnes Agées, Lome, Togo; 9 Université de Ouaga I, Ouagadougou, Burkina Faso

**Keywords:** HBs antigen, Risk factors, Serological profile, Lomé, Togo

## Abstract

**Background:**

In Togo, the prevalence of Hepatitis B Virus Surface Antigen (HBsAg) among young people aged 15–24 years was estimated at 16.4% in 2010; however, risk factors for HBsAg carriage are poorly documented. We sought to identify risk factors for HBsAg carriage and the serological profile of HBsAg carriers in Lomé (capital city of Togo).

**Method:**

We conducted a case control study from October 2016 to March 2017 in Lomé. Cases and controls were randomly selected from a database of Institut National d’Hygiène (INH) of Lomé during a free screening campaign for hepatitis B. We calculated means, frequencies, proportions, odds ratios (OR), and 95% confidence interval (CI) and performed logistic regression.

**Results:**

We included 83 confirmed cases and 249 controls. The median age was 31 years among cases and 30 years among the controls. The sex ratios (M/F) were 11/6 among cases and 4/3 for the controls. The independent risk factors for HBsAg carriage were the awareness of hepatitis B serological status (OR = 3.56, 95% CI [1.80–7.04]) and Kabyè-tem ethnic group (OR = 3.56, 95% CI [1.98–6.39]). Among HBsAg carriers, 13.3% were at the viral replication stage (all of whom were between 30 and 45 years of age) and 1.2% were at the acute stage of the disease. The prevalence of co-infection with hepatitis B and C was 4.80%. All co-infections were in women aged 24–28 years.

**Conclusion:**

The Kabyè-tem ethnic group is at risk of HBsAg carriage in Lomé. Of note, most HBsAg carriers in this ethnic group are aware of their HBsAg serological status. Furthermore, the prevalence of Hepatitis among adults of reproductive age is high and is cause for concern. We therefore recommend screening and vaccination campaigns at subsidized prices among people aged 30 years and older.

## Background

Hepatitis B is an inflammatory disease of the liver characterized by hepatocellular necrosis and caused by a virus belonging to the *Hepdnaviridae* family [1]. Hepatitis B is a major public health concern across the globe. The World Health Organization (WHO) estimated that approximately 240 million people are infected with the Hepatitis B Virus (HBV) worldwide [2] but only 11% of those who live with viral hepatitis are aware of their serologic status [3]. These individuals are at risk of severe illness and death as a result of cirrhosis or hepatocellular carcinoma (HCC). The viral hepatitis pandemic takes a heavy toll on lives, communities and health systems. In 2013, viral hepatitis was the seventh highest cause of mortality globally. It is responsible for an estimated 1.4 million deaths per year from acute infection and hepatitis-related liver cancer and cirrhosis a toll comparable to that of HIV and tuberculosis. Of those deaths, approximately 47% are attributable to HBV [4, 5]. The majority of hepatitis B infections are in Africa where the prevalence is estimated to be 7.2% (6.2–8.2) [6].

The virus is typically acquired via percutaneous or mucosal exposure to infected blood or other body fluids [7]. Because the virus can also be transmitted from mother to child during delivery and through unprotected sexual intercourse, infection of sexually active adults of reproductive age is particularly concerning. Acute HBV infection is characterized by the presence of hepatitis B surface antigen (HBsAg) and Immunoglobulin M (IgM) against Hepatitis B virus core antigen (anti-HBc IgM). During the initial phase of the infection, which is characterized by a high level of viral replication, the patients are carriers of the Hepatitis B viral antigen (HBeAg). The presence of HBeAg indicates that the subject is highly contagious. Antibodies against HBsAg (anti-HBs antibodies) appear a few weeks later, following which HBsAg is lost. Chronic infection is characterized by the persistence of HBsAg beyond 6 months [8]. The persistence of HBsAg is an important risk factor for development of chronic hepatitis and liver cancer.

A previous study estimated the prevalence of this disease in Togo to be 16.4% among young people (aged 15–24 years) in 2010 [9] it varied significantly with gender, marital status, place and region of residence. In that study, the prevalence was 19.2% among males and 13.9% among females. A study conducted in 2011 at University Hospital of Campus in Lomé found a prevalence of 19.08% with 25% in males and 14.8% in females [10]. In 2015, the estimated prevalence of HBsAg was 4.6% among first-year University students at the University of Lomé [11]. These prevalence figures are higher than the average recorded for the West African sub region highlighting the importance of this public health concern in Togo. Public health authorities have been progressively implementing measures to prevent hepatitis B infection since 2008, as evidenced by the integration of hepatitis B vaccination into the Expanded Program on Immunization (EPI) [12, 13]. A workshop on hepatitis B was organised in Lomé in November 2016. On this occasion, Institut National d’Hygiène of Lomé (INH) organized a free screening campaign during which 476 people were enrolled. Blood samples were collected from participants and tested for the presence of HbsAg with Enzyme Linked Immuno Sorbent Assay (ELISA). Samples which tested positive for HBsAg were further analyzed for HBeAg, anti-HBc IgM antibodies, and antibodies against Hepatitis C virus.

A database was established at the end of this campaign. In this study we present an analysis of that database, focusing on risk factors for HbsAg carriage and serological profiles of people with HbsAg, which are still poorly documented in Togo. The objectives of this study were to identify factors associated with carriage of HBsAg and to describe the serological profile of people with HBsAg in Lomé.

## Methods

### Study details

We conducted a case-control study at the INH of Lomé from October 2016 to March 2017. The study population consisted of individuals residing in Lomé who participated in a free screening campaign for HBsAg in October 2016. Information about the campaign was broadcasted on public radio and television to maximize the catchment of the campaign throughout the city of Lomé. A database containing serology data from the participants was built during this screening campaign. All participants were educated on the goals of the study and gave a written consent to participate. Prior to the study we obtained the approval of the INH internal ethics council.

### Terms and definitions

Case: Anyone who participated in the free HBsAg screening test at INH in October 2016 and tested positive on the ELISA HBsAg assay.

Control: Anyone who participated in the free HBsAg screening test at INH in October 2016 and tested negative on the ELISA assay HBsAg.

Active viral replication: Simultaneous presence of HBsAg and HBeAg.

Acute phase of hepatitis B: Simultaneous presence of HBsAg and anti-HBc IgM antibodies.

### Sampling and data collection

For a risk of 5%, a power of 80%, a case and control ratio of 1/3, a proportion of controls and cases with exposures of 40 and 57% respectively and an odd ratio of 2.1 we determined the minimal sample size to be 77 cases and 249 controls. We randomly selected controls and cases from records in database described in the study details. There were no exclusion criteria for the study. We gathered participant information through a questionnaire. In addition, we examined the laboratory register or the filled questionnaires to complete missing data and to delete duplications.

### Variables

We were interested in socio-demographic information such as age, sex, ethnic group, level of education, marital status, and place of residence. We also considered knowledge about hepatitis B transmission route and means of prevention. These variables are of interest because they may correlate with behavioural measures to avoid acquisition of the virus. In addition, we documented possible avenues of exposure such as prior blood transfusion, surgery, piercing, vaccination, dental care or surgery, hospitalization, and neighbours suffering of hepatitis B. Finally, we tracked serological markers: HBsAg, HBeAg, anti-HBc IgM antibody, and hepatitis C markers.

### Biological analysis

All blood samples were analysed for the presence of the HBsAg by means of the ELISA with Monolisa Kit from Biorad made in France. Samples which tested positive for HBsAg were further analysed for the presence of the HBeAg and anti-HBc IgM antibody by Electro-chemiluminescence immuno-assay (ECLIA) on Cobas machine from Roche and for the presence of Hepatitis C virus markers by ELISA with Monolisa Kit from Biorad.

### Data analysis

We completed the missing data, corrected the duplicates of the database and performed analyses using Epi Info 7. We calculated means, frequencies, proportions and measured associations by calculating the odds ratios (OR). We compared proportions by Fisher exact or Chi-square tests with a significance threshold of *p* ≤ 0.05.

Risk factors that were associated with HBsAg with *p* < 0.20 in bivariate analysis were introduced into a multivariable logistic regression model.

The explanatory variables were: previous history of vaccination, blood transfusion, hospitalization, surgery, dental care or surgery, ethnic group, sex, marital status, knowledge of HBV status, neighbour suffering of hepatitis B. The explained variable was the carriage of the HBsAg. We checked interactions of variables.

### Ethical considerations

Subjects enrolled in the free screening gave a written consent. Prior to the use of the screening database, we obtained clearance from the internal ethics council of INH of Lomé.

## Results

### Cases and controls characteristics

We included 83 cases and 249 controls in the study. Cases and controls had median ages of 31 years old (IQR 26–39 years) and 30 (IQR 23–41 years) respectively. Females represented 36.14% of cases and 43.5% of controls. Sex ratio (M/F) was 11/6 for cases and 4/3 for controls.

The characteristics of cases and controls are presented in Table 1.

**Table 1:** Characteristics of HBsAg-carrying cases and controls in Lomé, 2016

Characteristics	Cases n (%)	Controls n (%)
Age (years)		
0–15	0 (0.00)	30 (12.10)
16–30	32 (38.55)	90 (36.29)
31–45	43 (51.81)	76 (30.65)
46–60	7 (8.43)	40 (16.13)
> 60	1 (1.20)	12 (4.84)
Median age (years)	31	30
Sex		
Female	30 (36.14)	107 (43.50)
Male	53 (63.86)	139 (56.50)
Marital status		
Single	29 (35.37)	102 (49.51)
Married	51 (62.20)	99 (48.06)
Widowed/Divorced	2 (2.44)	5 (2.43)
Ethnicity		
Ewe	14 (17.28)	124 (52.54)
Kabyè-tem	49 (60.49)	70 (29.66)
Paragourma	13 (16.05)	22 (9.32)
Akposso-akébou	3 (3.70)	7 (2.97)
Ana-ifè	2 (2.47)	5 (2.12)
Other	0 (0.00)	8 (3.39)

### Bivariate analysis

Tables 2, 3 and 4 show the factors associated with the carriage of HBsAg in Lomé in bivariate analysis. Carriage of HBsAg was associated with ethnic group, age group, marital status, knowledge of the mode of contamination, and the knowledge of the hepatitis B serological status. No statistically significant association could be demonstrated between the carriage of HBsAg and previous hospitalization, surgery, dental care, tattooing, a neighbour suffering of Hepatitis B or vaccination.

**Table 2:** Socio-demographic risk factors for carriage of HBsAg in Lomé, 2016

Socio-demographic factors	Cases n (%)	Controls n (%)	OR	95% CI	*p*-value
Sex					
Male	53 (63.86)	139 (56.50)	1.36	0.81–2.27	0.15
Female	30 (36.16)	107 (43.50)			
Age					
≤ 30 years	81 (97.59)	209 (84.27)	7.56	1.78–32.03	**< 0.001**
> 30 years	2 (2.41)	39 (15.73)			
Marriage status					
Yes	51 (62.20)	99 (48.06)	1.78	1.05–3.00	**0.04**
No	31 (37.80)	107 (51.94)			
Kabyè-tem ethnic group					
Yes	49 (60.49)	70 (29.66)	3.63	2.14–6.14	**< 0.001**
No	32 (39.51)	166 (70.34)

**Table 3:** Risk factors for carriage of HBsAg related to history of medical and surgical interventions in Lomé, 2016

Risk factors	Cases n (%)	Control n(%)	OR	95 %CI	p
History of surgery					
Yes	10 (12.05)	34 (13.65)	0.87	0.41–1.84	0.43
No	73 (87.95)	215 (86.35)			
History of Hospitalisation					
Yes	23 (27.71)	78 (31.33)	0.84	0.48–1.45	0.84
No	60 (72.29)	171 (68.67)			
Dental care or surgery					
Yes	13 (15.66)	37 (14.86)	1.06	0.54–2.12	0.49
No	70 (84.34)	212 (85.14)			
Blood transfusion					
Yes	6 (7.23)	10 (4.03)	1.85	0.65–5.27	0.18
No	77 (92.77)	238 (95.97)			
Vaccination					
Yes	2 (2.41)	18 (7.26)	0.315	0.07–1.39	0.08
No	81 (97.59)	230 (92.74)

**Table 4:** Risk factors for carriage of HBsAg related to knowledge of HBV infection and certain practices in Lomé, 2016

Risk factors	Case n(%)	Control n(%)	OR	95%CI	p
Knowledge of hepatitis B Virus transmission					
Yes	59 (71.08)	143 (58.85)	1.71	1.00–2.95	0.03
No	24 (28.92)	100 (41.15)			
Awareness of Hepatitis B serological status					
Yes	30 (36.14)	35 (14.11)	3.44	1.94–6.11	< 0.001
No	53 (63.86)	213 (85.89)			
Tattoo					
Yes	3 (3.61)	7 (2.81)	1.30	0.33–5.13	0.47
No	80 (96.39)	242 (97.19)			
Neighbour suffering of Hepatitis B					
Yes	15 (18.29)	30 (12.05)	1.63	0.83–3.2	0.19
No	67 (81.71)	219 (87.95)			

### Multivariable analysis

Being of the Kabyè-tem ethnic group and knowing its HBV serological status were demonstrated as independent risk factors for carrying HBsAg as shown in Table 5. Vaccination did not appear to have a statistically significant protective effect.

**Table 5:** Independent Risk Factors for Carriage of HBsAg in Lomé, 2016

Risk factors	Odds Ratio	95%CI		*p*-Value
Age > 30 years	1.67	0.33–8.55		0.54
Kabyè-tem	3.56	1.98–6.39		0.001
Marriage	1.44	0.78–2.64		0.24
Knowledge of hepatitis B Virus transmission	1.07	0.56–2.06		0.84
Male sex	1.42	0.78–2.59		0.26
Awareness of HBV serological status	3.56	1.80–7.04		< 0.001
Neighbors with HBV	1.47	0.63–3.43		0.37
Blood transfusion	1.90	0.58–6.27		0.29
Vaccination	0.27	0.06–1.26		0.10

### Serological profile of HBsAg carriers

As shown in Table 6, 13.3% of HBsAg carriers were at the stage of active viral replication (i.e. carry HBeAg), and 1.2% were in the acute phase or in activation (i.e. carry the anti-HBc IgM antibody), while 4.8% had markers of hepatitis C virus infection. The median age of the hepatitis B cases which were categorized as being in the stage of viral replication was 32.27 years (IQR 25–40 years), the sex ratio (M / F) =3/4. All patients in this category belong to the Kabyè-tem ethnic group. Additional features of persons in viral replication are presented in Table 7. HBV and HCV co-infected people were all female and aged 24–28 years and belong to Kabyè-tem ethnic group. Participants in this subgroup were also unaware of their status and did not know the routes of transmission or methods for prevention of HBV.

**Table 6:** Serological profile of people carrying HBsAg in Lomé 2016 (*n* = 83)

Serologic status	Frequency (n)	Percentages (%)
Ag HBe	11	13.30
anti HBc IgM antibody	1	1.20
Hepatitis C	4	4.80

**Table 7:** Characteristics of persons carrying HBsAg at viral replication stage in Lomé, 2016 (*n* = 11)

Characteristics	Frequency	Percentage (%)
Sex		
Male	5	45.45
Female	6	54.55
Marital status		
Single	6	54.55
In couple	3	27.27
Not specified	2	18.18

## Discussion

The Kabyè-tem ethnic group membership and knowledge of the serological status before the test, were identified as two independent risk factors associated with HBsAg carriage. Of the infected persons, 13.3% were still infectious, and these were all women from the Kabyè-tem ethnic group. Our findings corroborate the results of a previous study that observed that the regions of highest HBsAg seroprevalence among 15–24 years old individuals were the central region (27.7%) and the Kara region (23.1%), which are inhabited by the Kabyè-tem [9]. This ethnic group is the second largest in Togo. Although Kabyè-tem primarily live in the central and Kara regions, many have moved to reside in Lomé and were thus included in this study. To our knowledge, there has not been a study comparing the socio-economic conditions of members of the Kabyè-tem ethnicity relative to other ethnicities. That belonging to the Kabyè-tem group is an independent risk factor for HBsAg carriage could be explained by socio-cultural or genetic factors. Several studies [14, 15] have described the influence of genetic factors on HBV infection and on the elimination of HBsAg.

The fact that knowledge of serological status was shown to be an independent risk factor indicates that most cases were aware of their serological status compared to controls and that screening for HBsAg was not routinely done in Togolese populations. The cases were probably diagnosed with hepatitis B during clinical manifestations prior to our survey. Statistically significant associations could not be identified between HBsAg carriage and the male gender, transfusion, hospitalization, surgery, tattoos and piercing, or having a parent suffering from hepatitis B. Similar observations were made in a previous study conducted in Nigeria in 2011 [16] and another conducted in Ghana in 2013 [17]. This may be related to the fact that this information was obtained on the basis of statements from study participants, they were not likely willing to disclose this information because of stigma. Also, the sample size was too small for identification of certain risk factors such as transfusion, prior hospitalization, or dental surgery. In addition, it is possible that the fact that blood transfusions, hospitalization, and nursing practices are becoming increasingly safe [2] minimized the rate of hepatitis acquisition via iatrogenic routes.

In the hospital-based Lomé study and the national survey among people aged 15 to 24 years there was a statistically significant difference in the prevalence of HBsAg between the two genders [9, 10]. This discrepancy may be due to our smaller sample size. We also found that vaccination did not have a statistically significant protective effect, this possibly reflects a poor adherence to vaccination schedules.

Of HBsAg carriers, 13.3% also carried HbeAg and were therefore still contagious. Importantly, this population is likely sexually active (median age 28 years) and therefore at risk of transmitting the virus to uninfected individuals [18]. A similar prevalence (13.7%) was found in a study carried out among PLHIV on ART in Lomé in 2011 [19], indicating that the odds of sexual transmission in Lomé have remained high for some time.

The prevalence of hepatitis B and C co-infection was 4.8%, which was higher than the 3.9% value previously reported among untreated HIV patients in Tanzania during 2006 [20]. It is also much higher than the 0.6% previously reported in Ghana among pregnant women in the Ashanti region [17]. It therefore appears that co-infection is a significant issue in Togo.

## Limitations

Three major limitations were identified. The first limitation is that our study did not incorporate risk factors such as unsafe sexual behaviour and type of breastfeeding. The second was the fact that the serological diagnosis of the hepatitis B we used in our study may have not detected occult hepatitis whose prevalence is approximately 5% among HBsAg negative individuals [21, 22]. Our study may therefore have underestimated the association with risk factors. The third was that the pre-existing database from which we used unfortunately, did not include information regarding sexual practices and intravenous drug use by the participants; these important risk factors will be addressed in future studies. Despite these limitations, analysis of this database allowed us to identify factors associated with HBsAg carriage and the serological profile of HBsAg carriers in Lomé in 2016.

## Conclusion

Our study showed that Kabyè-tem ethnic group and awareness of hepatitis B serological status are independent risk factors for HBsAg carriage in Lomé. A proportion of 13% of HBsAg carriers in Lomé, were in the contagious phase, implying that the risk of transmission of this disease to sexual partners and close neighbours (parents, nurses, domestic workers) remains high in Togo. Education campaigns to encourage screening and subsidization of the vaccination especially among the Kabyè-tem community (particularly women) will likely promote effective control of hepatitis B in Togo. The Expanded Program on Immunization should be reviewed in order to incorporate introduction of Hepatitis B vaccine earlier during the first hours of life to minimize the risks of mother to child transmission.

## Abbreviations

ART: Anti-Retroviral Treatment
CHU: Centre Hospitalier Universitaire
CI: Confidence interval
ECLIA: Electro-chemiluminescence immuno-assay
ELFA: Enzyme linked fluorescent assay
ELISA: Enzyme Linked Immuno Sorbent Assay
EPI: Expanded Program on Immunization
HBeAg: e Antigen of hepatitis B virus
HBsAg: Surface antigen of hepatitis B virus
HBV: Hepatitis B virus
HCC: Hepatocellular carcinoma
IgG: Immunoglobulin gamma
IgM: Immunoglobulin Mu
INH: Institut National d’Hygiène
IU: International unit
OR: Odd ratio
PCR: Polymerase chain reaction
PLHIV: Person living with HIV
WHO: World Health Organisation
